# SR proteins in cancer: function, regulation, and small inhibitor

**DOI:** 10.1186/s11658-024-00594-6

**Published:** 2024-05-22

**Authors:** Mingrong Bei, Jianzhen Xu

**Affiliations:** 1 Systems Biology Laboratory, Shantou University Medical College (SUMC), 22 Xinling Road, Shantou, 515041 China; 2 Department of Cardiology, First Affiliated Hospital of Shantou University Medical College, Shantou, China

**Keywords:** SR proteins, RNA processing, Splicing, m^6^A modification, Tumor treatment

## Abstract

Alternative splicing of pre-mRNAs is a fundamental step in RNA processing required for gene expression in most metazoans. Serine and arginine-rich proteins (SR proteins) comprise a family of multifunctional proteins that contain an RNA recognition motif (RRM) and the ultra-conserved arginine/serine-rich (RS) domain, and play an important role in precise alternative splicing. Increasing research supports SR proteins as also functioning in other RNA-processing-related mechanisms, such as polyadenylation, degradation, and translation. In addition, SR proteins interact with N^6^-methyladenosine (m^6^A) regulators to modulate the methylation of ncRNA and mRNA. Dysregulation of SR proteins causes the disruption of cell differentiation and contributes to cancer progression. Here, we review the distinct biological characteristics of SR proteins and their known functional mechanisms during carcinogenesis. We also summarize the current inhibitors that directly target SR proteins and could ultimately turn SR proteins into actionable therapeutic targets in cancer therapy.

## Introduction

**Fig. 1:**
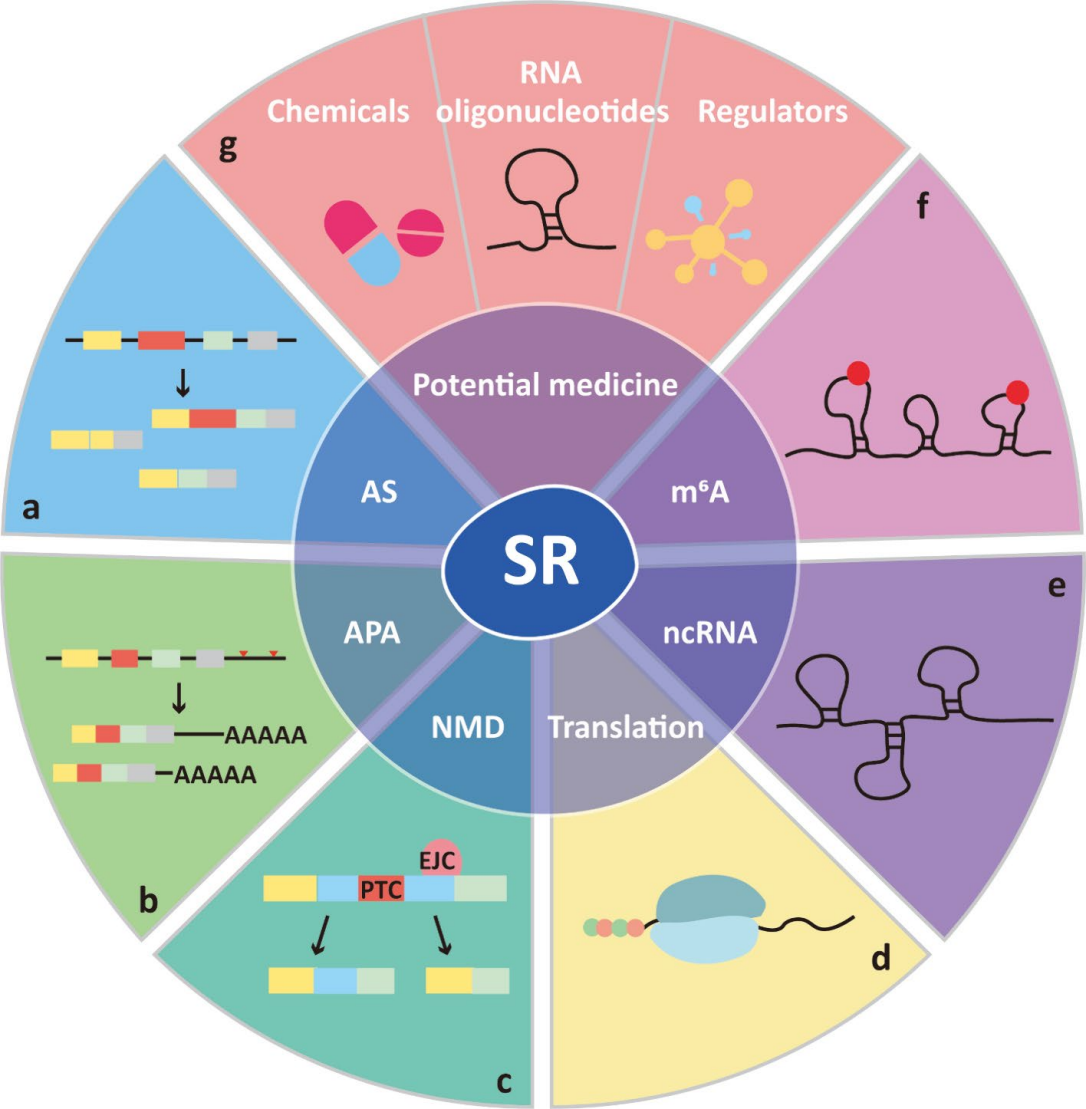
The multifaceted role of SR proteins and their direct targeted inhibitors for cancer treatment. **a** SR proteins regulate the alternative splicing of pre-mRNA and contribute to the diversity of the transcriptome and proteome. **b** SR proteins regulate alternative cleavage and polyadenylation to generate distinct 3′ ends of mRNAs and ncRNA. **c** SR proteins regulate nonsense-mediated decay to prevent the translation of potentially deleterious truncated proteins. **d** SR proteins regulate mRNA translation. **e** SR proteins interact with noncoding RNA to regulate biological processes. **f** SR proteins participate in N^6^-methyladenosine modification. **g** Potential medicines directly target SR proteins, including chemicals, RNA oligonucleotides, and SR protein regulators

Serine and arginine-rich proteins (SR proteins) are conserved RNA-binding proteins present in all plants and metazoans [1]. SR proteins are involved in constitutive and alternative splicing (AS) of pre-mRNA, which is one of the most important steps during RNA processing, and contributes to the diversity of the transcriptome and proteome [2, 3]. Apart from this canonical role, SR proteins have noncanonical roles in RNA regulation by participating in alternative cleavage and polyadenylation (APA), nonsense-mediated decay (NMD), mRNA export, mRNA translation, and interaction with ncRNA [4, 5]. In particular, SR proteins also participate posttranscriptionally in RNA N^6^-methyladenosine (m^6^A) modification of various RNAs by directly or indirectly interacting with methyltransferases [5] (Fig. 1).

SR proteins are critical to metazoan development. Inactivation of serine and arginine-rich splicing factor 1 (SRSF1) and SRSF6 causes embryonic lethality in chickens and Drosophilae [6]. In addition, SRSF1, SRSF2, SRSF3, SRSF5, and transformer 2 beta homolog (TRA2B) have been reported to play a pivotal role in heart, skeletal muscle, liver, and central nervous system development in the corresponding gene knockout mice [7–11]. On the other hand, dysregulation of SR proteins also contributes to tumorigenesis and metastasis. For example, the generation of oncogenic isoforms of SRSF1, SRSF3, SRSF6, and TRA2B have been associated with lung cancer, breast cancer, and colon cancer [12]. These cancer-related isoforms can enhance cell escape from apoptosis and confer drug resistance [13]. Therefore, SR proteins act as pivotal regulators to affect RNA metabolism and gene expression in tumor cells. In the present review, we summarize the diverse functions of SR proteins and their characteristics, as well as the therapeutic compounds and RNA oligonucleotides that directly target SR proteins in the treatment of cancer (Fig. 1).

## Biochemical characteristics of SR proteins

SRSF1 was first identified in the early 1990s, and was the first SR protein found to be involved in preventing exon skipping and ensuring splicing accuracy in AS [14, 15]. Intensive studies on RNA splicing have subsequently shown that other SR proteins (i.e., SRSF2-12) take part in splicing complementation [16–22]. These classical SR proteins are characterized by one or two RNA recognition motifs (RRM) at the N-terminus, and an RS domain, rich in serine/arginine dipeptide repeats, at the C-terminus [1]. Remarkably, two TRA family members, TRA2A and TRA2B, are now regarded as SR-like proteins. They have only one RRM domain but two RS domains, and function as sequence-specific splicing activators [23]. TRA2A is functionally conserved because human TRA2A can replace its *Drosophila* homolog to affect both female sexual differentiation and AS of *dsx* pre-mRNA [24]. TRA2B has been reported to stimulate full-length survival motor neuron 2 (SMN2) expression through its ability to regulate AS [25].

Among the 14 known SR proteins, SRSF1, SRSF4, SRSF5, SRSF6, and SRSF9 have one canonical RRM and one pseudo-RRM [26]. The main role of RRMs is to recognize specific pre-mRNA binding sites and dictate the position of SR proteins on RNA sequences [1]. A recent study has reported that the pseudo-RRM of SRSF1 frequently competes with splicing repressors, such as hnRNPA1, rather than recruiting spliceosomal components to regulate splicing [27]. Structural NMR studies revealed that the conserved residues located in α-helix 1 of the pseudo-RRM contribute to recognition of the specific GGA motif in pre-mRNA, and this unusual mode of RNA recognition is conserved in all pseudo-RRMs [27]. In addition, the RS domain of SR proteins is conserved across vertebrates and invertebrates, and coordinates protein–protein or protein–RNA interactions by phosphorylating serine residues [28]. SR protein kinases, including SR protein kinases (SRPKs), Cdc2-like kinases (CLKs), and dual-specificity tyrosine-regulated kinases (DYRKs), have been shown to be specifically responsible for catalyzing the numerous serine residue phosphorylations on the RS domain [29–31]. The RS domain phosphorylation/dephosphorylation state is important for two nuclear import receptors, transportin-SR1 (TRN-SR1) and transportin-SR2 (TRN-SR2) to target SR proteins to the nucleus, thereby affecting their cellular distribution and functional flexibility [32–34]. It has been reported that TRN-SR2 imports TRA2B only when it is phosphorylated, but its splice variant TRN-SR1 can import both unphosphorylated and phosphorylated TRA2B [35]. Moreover, phosphorylation of the RS domain is necessary for SR proteins to leave nuclear speckles and bind to RNA, whereas a hypophosphorylated RS domain is required for SR protein splicing activity and the transport of RNA from the nucleus to cytoplasm with the help of the TAP/NFX1 nuclear export receptor [36, 37]. Dephosphorylation of the RS domain also promotes cytoplasmic mRNA binding to SR proteins and enhances SR proteins in translational activity [38]. In addition to its RRM and RS domains, SRSF7 has a unique CCHC-type zinc finger domain, which is thought, together with the RRM domain, to confer RNA-binding specificity [23, 39].

## Functional mechanisms of SR proteins in cancer

Essential roles of SR proteins have been reported for sex determination [40, 41], cell differentiation [42, 43], development of the brain [44, 45] and heart [10, 46, 47], the immune system [48, 49], and many types of cancer [12, 50, 51]. Here, we focus on the known functional mechanisms of SR proteins, and discuss the importance of these functions in various cancers.

### Regulation of constitutive and alternative splicing of pre-mRNAs by SR proteins

**Fig. 2:**
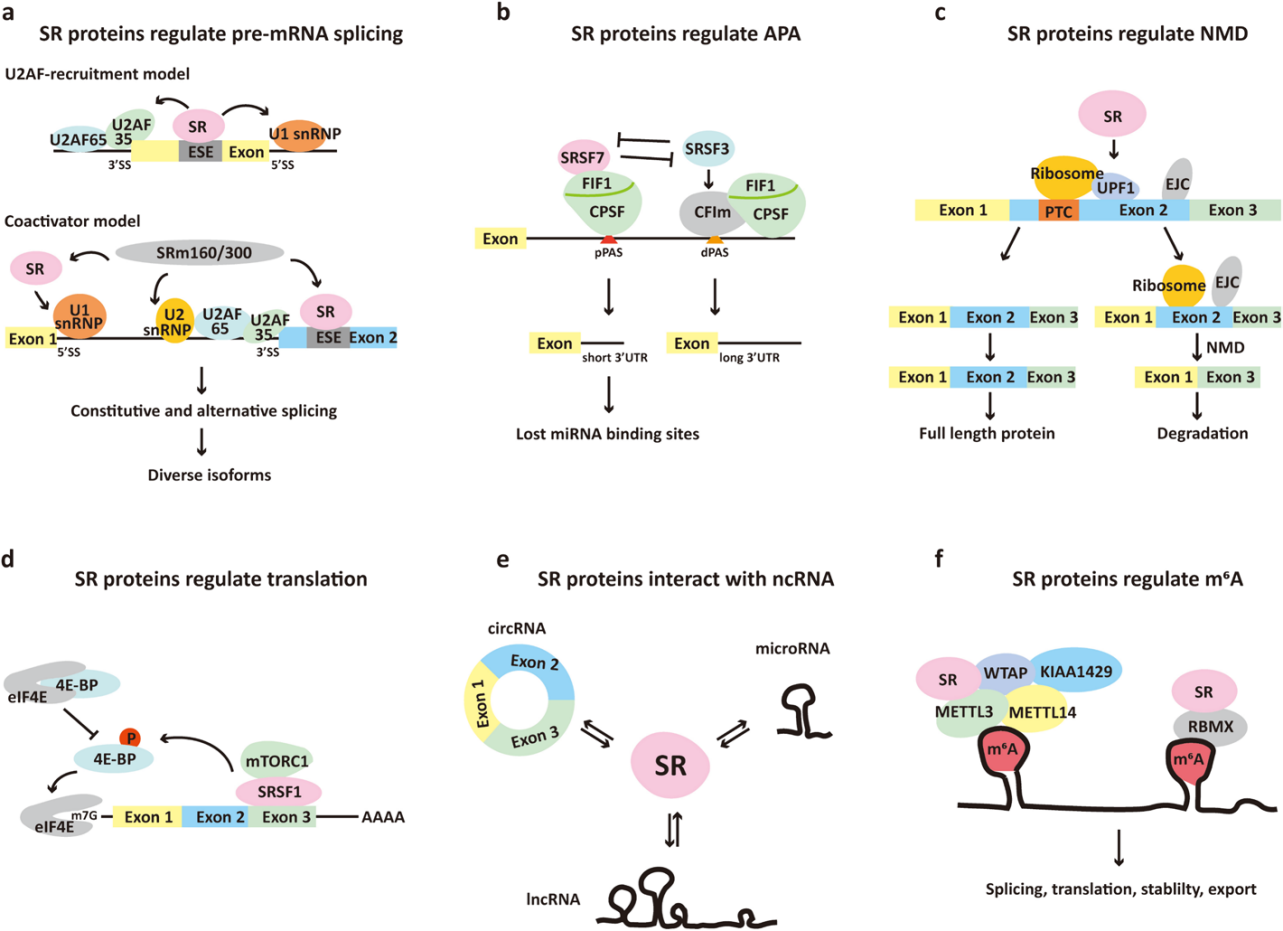
Mechanisms of SR proteins in human cancers. **a** Two models of SR protein function in splicing. U2AF-recruitment model: ESE-bound SR proteins interact with U2AF35 at the 3′SS to stabilize the binding of U2AF65 at the polypyrimidine tract and recruit U1 snRNP at the 5′SS to active splicing. Coactivator model: ESE-bound SR proteins interact with splicing coactivator SRm160/300 and communicate with U1 snRNP and U2 snRNP. **b** Model of SRSF3- and SRSF7-mediated regulation of APA by interacting with the APA component on PASs in an antagonistic manner. **c** Model of SR proteins recognizing NMD factor UPF1 downstream of the PTC to activate the NMD pathway, thus inducing protein degradation. **d** Example of SR protein function in translation. SRSF1 recruits mammalian target of rapamycin complex 1 (mTORC1) to mRNA to promote phosphorylation of 4E-BP, leading to release of eIF4E and initiation of translation. **e** SR proteins can interact with lncRNA, circRNA and microRNA to regulate tumorigenesis. **f** SR proteins can directly or indirectly interact with m^6^A methyltransferases, or colocalize with m^6^A readers to modulate m^6^A modification

Constitutive and alternative splicing of pre-mRNA is a crucial part of eukaryotic gene expression in metazoans, and contributes to proteomic by enabling a single gene to encode multiple different transcripts with distinct functions [52, 53]. Nuclear pre-mRNA splicing is catalyzed by a macromolecular ribonucleoprotein (RNP) complex termed the spliceosome [54]. Spliceosomes recognize the four major regulatory sequences in pre-mRNA introns, including the 5′ splice site (5′SS), the intron branch point site (BP), the 3′ splice site (3′SS), and the polypyrimidine tract [55]. SR proteins serve as molecular adapters to assemble spliceosomes and produce diverse pre-mRNAs in the splicing of transcripts [1]. Two major models have been proposed to explain the mechanism by which SR proteins affect cis-regulatory elements, called exonic splicing enhancers (ESEs), on pre-mRNAs to regulate exon inclusion (Fig. 2a). The “U2AF-recruitment” model involves SR proteins binding to an ESE to interact with U2AF35 at the 3’SS to stabilize the binding of U2AF65 at the polypyrimidine tract. Simultaneously, SR proteins also recruit and stabilize U1 snRNP at the 5′SS to activate splicing in a process known as exon definition [3, 56]. The “coactivator” model proposes that ESE-bound SR proteins can interact with basal components of the spliceosome through a bridging factor named the SRm160/300 splicing coactivator, or communicate with U1 snRNP and U2 snRNP [56, 57]. Abnormal expression of SR proteins are found in almost all tumor types, and results in dysregulated RNA splicing and tumor progression [12].

SRSF2 is involved in the switch in AS of various pre-mRNAs for apoptotic genes, such as caspase-8, caspase-9 and Bcl-x, to favor proapoptotic splice variants in lung carcinoma [58]. Similarly, downregulation of SRSF2 in renal tumors results in concomitant changes of splicing profiles of apoptosis-associated genes by decreasing the expression of proapoptotic isoforms caspase-9a, Smac3, Surv-2B, BimS, Bimα3, and MCL-1S, while increasing the expression of anti-apoptotic isoforms caspases-9b and caspase-8L, therefore leading to cell proliferation [59]. Furthermore, the low level of SRSF3 in relapsed B-cell acute lymphoblastic leukemias inhibits the retention of exon 2 in CD19, causing failure to trigger killing by chimeric antigen receptor-armed T cells (CART-19), which leads to resistance to CART-19 immunotherapy [60]. These findings show anti-tumor roles of SR proteins by AS. In contrast, in breast cancer, overexpressed SRSF1, through its function in constitutive and alternative splicing, increases BIN1 isoforms that lack pro-apoptotic functions, thereby causing BIN1 failed interaction with MYC, and leading to MYC-induced epithelial cell transformation [61]. In addition, upregulated TRA2A in triple-negative breast cancer (TNBC) promotes the generation of biologically inactive RSRC2, which retains exon 4 in its transcript, thus contributing to the migration, invasion, and paclitaxel resistance of TNBC cells [62]. Overall, the above studies indicate that SR proteins play a dual role in different types of cancers by regulating pre-mRNA constitutive and alternative splicing.

### Regulation of alternative cleavage and polyadenylation by SR proteins

Alternative cleavage and polyadenylation is a widespread RNA-processing mechanism across all eukaryotic species and refers to the regulated selection of polyadenylation sites (PASs) to generate distinct 3′ ends on RNA polymerase II transcribed RNAs, including mRNAs and ncRNA [63]. APA frequently occurs in the 3′ untranslated region (3′UTR) of mRNAs, thus producing multiple mRNA and protein isoforms derived from a single gene to regulate RNA stability and facilitate RNA nuclear export [63]. APA is initiated by the APA machinery assembling from four elements at each PAS, comprising cleavage stimulatory factor (CSTF), cleavage and polyadenylation specificity factor (CPSF), cleavage factor Im (CFIm), and cleavage factor IIm (CFIIm) [64]. SRSF3 and SRSF7 have been reported to regulate APA processing by interacting with APA components on PASs in an antagonistic manner in an embryonic carcinoma cell line [65] (Fig. 2b). Specifically, SRSF7 interacts with FIP1, a subunit of CPSF, independently of RNA via its hypophosphorylated RS domain to activate proximal PAS (pPAS) usage directly in a splicing-independent manner, thus promoting short 3′UTRs. Conversely, SRSF3 controls the levels of active CFIm, which enhances cleavage at distal PASs (dPASs) and directly counteracts SRSF7 levels to promote long 3′UTRs by inhibiting pPAS usage [65]. The distinct function between SRSF3 and SRSF7 is owing to the unique domain present in SRSF7, which containing a stretch of 27 amino acids enriched in hydrophobic residues and a Zn knuckle, which are absent in SRSF3 [65]. It has been widely confirmed that mRNA isoforms with shorter 3′UTRs lose microRNA-mediated repression, therefore increasing oncogene RNA stability and translation to promote tumorigenesis [66–68]. In addition, SRSF3 and SRSF7 are both reported to connect polyadenylation with NXF1-mediated mRNA export, therefore regulating the transcripts with alternative 3′ end export from the nucleus to the cytoplasm [69].

### Regulation of nonsense-mediated decay by SR proteins

Nonsense-mediated decay is regarded as a cellular surveillance mechanism to promote degradation of erroneous transcripts containing premature termination codons (PTCs), thus preventing the translation of potentially deleterious truncated proteins [70]. One major molecular mechanism involved in the NMD pathway depends on the formation of a key regulatory player called the exon junction complex (EJC) [71]. An EJC is deposited ~20–24 nucleotides upstream of most exon–exon junctions and is removed from the mRNA by ribosomes during translation. In certain transcripts, ribosomes stop at the PTC, resulting in EJCs remaining downstream of the PTC, allowing recognition of EJCs by NMD factors to activate NMD [72] (Fig. 2c). NMD is an important biological mechanism of RNA processing, partially regulated by SR proteins in tumor development. In HeLa cells, SRSF1 promotes the recruitment of EJC factors and UPF1 to downstream of a PTC by its RS domain to elicit NMD [73]. In addition, an SRSF2 Pro95 mutation enhances the deposition of EJCs downstream of PTCs, associating with key NMD factors to enhance mRNA decay in acute myeloid leukemia [74].

Interestingly, owing to a highly ultraconserved PTC known as a poison exon (PE), SR proteins not only regulate other genes, they usually participate in their own NMD regulation. For example, binding of TRA2B to *TRA2B-PE* enhances *TRA2B-PE* inclusion, thus generating a transcript that cannot be translated [75]. Conversely, knockdown of SRSF3 has been shown to contribute to *SRSF3-PE* inclusion [76]. In addition, it has been reported that SRSF1 negatively regulates *SRSF4-* and *SRSF11-PE* inclusion, and positively regulates *SRSF2-*, *SRSF3-*, *SRSF6-*, *SRSF7-*, and *TRA2B-PE* inclusion [76]. SRSF4 and SRSF5 both regulate *SRSF6-PE* inclusion positively [76]. These cross-regulations and/or autoregulations among SR proteins via the NMD pathway precisely control the balance of their different spliced isoforms. Thus, the role of the SR proteins in RNA processing not only depends on their phosphorylated state, but also relies on their total protein level, distinct isoform levels and protein ratio between the cytoplasm and nucleus. In this respect, systematic dissection of the specific roles of each SR protein and the precise mechanism to control its expression in different cells may help us clarify the importance of each SR protein in cancer. Since SR proteins cross-regulate shared target genes, including the transcripts of each other, and form a dense coordinated network [76], a systems biology approach can be applied to globally identify the most dysregulated SR protein–RNA circuits under cancer conditions. This complementary approach enables the researcher to simultaneously characterize all alterations in SR proteins and to balance their multifunctionality without being trapped into negligible details [77, 78].

### Regulation of translation by SR proteins

The continuous shuttling of SR proteins between the nucleus and cytoplasm not only plays a role in RNA transcription, but also influences the regulation of mRNA translation. SRSF1 has been reported to take part in cap-dependent translation. Specifically, SRSF1 can bind to specific mRNAs that contain SRSF1-binding sites and serve as an adapter protein to recruit mTORC1 to the target mRNA by its second RRM domain and promote eIF4E-binding protein (4E-BP) phosphorylation, leading to the release of cytoplasmic cap-binding protein eIF4E and eliciting the activation of translation [79] (Fig. 2d). Additionally, SRSF1 was found to cosediment with the 80S ribosome and polysomes to promote translation in an RS domain-dependent manner [80]. Other cytoplasmic SR proteins also function in mRNA translation. For instance, SRSF1 and SRSF9 can bind to β-catenin mRNA and enhance its protein synthesis in a mTOR-dependent manner to promote β-catenin accumulation, thus mediating tumorigenesis [81]. SRSF10 interacts with the peptidyl transferase center of 28S rRNA to regulate ribosome biogenesis and translation [82]. On the contrary, hypoxia-induced SRSF3 promotes specific retention of intron 12 in EIF2B5, resulting in EIF2B5 protein decrease by the NMD pathway; therefore, inhibiting overall initiation of translation to protect head and neck cancer cells from extreme hypoxia, ensuring cell survival [83]. In addition, SRSF3 interacts with the 5′UTR of programmed cell death 4 (PDCD4) mRNA to repress translation and recruit PDCD4 mRNA to P-bodies for mRNA silencing [84]. Furthermore, SRSF7 can bind to its own pre-mRNA to promote inclusion of a PE and cause NMD-mediated transcript degradation to autoregulate itself at the translational level, thus modulating protein homeostasis during carcinogenesis [85].

### SR proteins interact with noncoding RNA

SR proteins also interact with a variety of ncRNA, including lncRNA, circRNA, and microRNA, to regulate biological processes (Fig. 2e). Metastasis-associated lung adenocarcinoma transcript 1 (MALAT1) is a typical lncRNA that has been widely reported to associate with SR proteins to modulate downstream pathways. MALAT1 localizes to nuclear speckles and interacts with the RRM domain of SR proteins. MALAT1 acts as a regulator of the phosphorylation/dephosphorylation status of SR proteins, contributing to alter SR protein-associated AS modulation, NMD pathway, and translation [36]. In breast cancer cells, SRSF1 interacts with mutant p53 and ID4 in a MALAT1-dependent manner. Mutant p53 and ID4 proteins promote the stabilization of SRSF1 binding of to MALAT1, thus inducing an increase of proangiogenic VEGFA isoforms while inhibiting the production of anti-angiogenic VEGFA isoforms [86]. Additionally, Pushkar et al. have reported that overexpression of MALAT1 in hepatocellular carcinoma activates the transcription of SRSF1, inducing the shorter spliced variant of S6K1, called Iso-2, an oncogene that can activate mTORC1 and induce increased 4E-BP phosphorylation [87]. SR proteins also interact with circRNA. For instance, SRSF10 can bind to the back-splice junction of cTTN1, a class of RBM20-dependent circRNAs that collectively regulate targets downstream of SRSF10 [88]. In colorectal cancer, the tumor promoter circPLCE1 directly binds to SRSF2, resulting in the repression of SRSF2-dependent PLCE1 pre-RNA splicing, which leads to tumor progression [89]. Furthermore, CLIP-seq analysis has shown that microRNAs associate with SR proteins [90], though the precise mechanism is still unknown. One possibility is that circRNAs work as a sponge for microRNA and protein, offering an opportunity for microRNAs to compete with SR proteins for binding to circRNA, thus modulating downstream molecular events, such as RNA splicing or RNA export. Overall, the study of SR protein–ncRNA interaction is still in the early stage and needs to be further investigated.

### SR proteins involvement in N^6^-methyladenosine modification

N6-methyladenosine (m^6^A) is one of the most common reversible modifications in eukaryotic RNAs [91]. Regulation of m^6^A modification plays an essential role in RNA metabolism, including mRNA decay, pre-mRNA splicing, mRNA export, translation regulation, and ncRNA processing [92]. Several SR proteins have been reported to participate in m^6^A modification, thereby controlling RNA fate (Fig. 2f). For example, Zu et al. have shown that the upregulation of SRSF3 in pancreatic cancer cells contributes to increased m^6^A modification on lncRNA ANRIL [93]. The m^6^A decorations on ANRIL are responsible for SRSF3 binding to ANRIL and generating the ANRIL-L isoform via AS. ANRIL-L associates with another two proteins, Ring1B and EZH2, to form a complex to enhance drug resistance and DNA homologous recombination repair [93]. Furthermore, SRSF3, through both its RRM and RS domain, was also found to directly bind to the m^6^A reader YTHDC1 and the mRNA export receptor NXF1 to facilitate the export of m^6^A methylated mRNAs [94, 95]. In addition, SRSF7 colocalizes with the methyltransferase complex containing METTL3, METTL14, and WTAP in the nucleus; knockdown of SRSF7 decreases the m^6^A modification near the SRSF7 m^6^A modification binding sites on mRNA. SRSF7 modulates mRNA m^6^A methylation through recruiting METTL3, independent of its canonical role in AS and APA, to promote proliferation and migration of glioblastoma cells [96]. Recently, we have demonstrated that TRA2A induces esophageal cancer progression via MALAT1 [97]. Mechanistically, TRA2A directly interacts with core methyltransferase METTL3 and the m^6^A reader RBMX to regulate the methylation and stability of MALAT1 [98].

As we describe above, SR proteins participate in AS, APA, NMD, mRNA translation, ncRNA interaction, and m^6^A modification, so the mystery is how SR proteins accurately engage their function in a specific process, or how they precisely switch between their canonical and noncanonical roles during RNA processing. In eukaryotes, m^6^A methylation has been shown to be involved in almost the entire RNA life cycle, including splicing, translation, degradation, and transportation [99, 100]. Thus, we postulate that SR proteins may be guided by the m^6^A marks in RNA to dynamically execute their function to comprehensively regulate RNA metabolism. Indeed, Zhao et al. have reported that m^6^A sites are enriched in exonic regions flanking 5′- and 3′-splice sites, that overlap with the ESE binding regions, suggesting that m^6^A signals may be related to the activity of SR proteins [101]. Further supporting this point, we and others recently found that several SR proteins, including TRA2A, SRSF3, and SRSF7, can interact with METTL3 and other proteins in RNA methylation, and the m^6^A signal in pre-mRNA is essential for SR proteins to participate in RNA processing [94, 96, 98, 102].

## Designing drugs to target SR–RNA interactions for clinical use

As mentioned above, phosphorylation of the SR protein RS domain controls the localization and activity of SR proteins. Much effort has been spent in the development of small molecular inhibitors to target SR protein-associated kinases. Dozens of compounds have been discovered and have demonstrated promising antitumor activity in vivo. Interested readers are referred to more extensive reviews for available inhibitors currently being assessed in preclinical/clinical studies [103–105]. Here, we mainly focus on inhibitors directly targeting the SR proteins themselves by means of chemicals and RNA oligomers (Table 1).

**Table 1:** Chemicals and RNA oligonucleotides inhibiting SR proteins

RNA oligos	SR protein	Drug	Target RNA	Mechanism	Cancer type	PMID
Small molecule inhibitor	SRSF3	SFI003	DHCR24/ROS axis	Disrupt SRSF3-mediated splicing by reducing its protein level	Colorectal cancer	35501301
	SRSF6	Indacaterol	ZO-1 and ECM1	Affect SRSF6-mediated splicing by binding to RRM2	Colorectal cancer	29114070
	TRA2A	Nebivolol	MALAT1	Compete with MALAT1 to bind TRA2A	Esophageal cancer	37317053
Splice switching oligos	TRA2B	Antisense oligonucleotide	TRA2B	Promote TRA2B poison exon inclusion and inhibition of TRA2B protein expression	Breast cancer	33176162
	SRSF3	Antisense oligonucleotides	SRSF3	Promote the inclusion of exon 4 to reduce SRSF3 expression	Oral cancer	35540349
shRNA or siRNA	TRA2A	siRNA	TRA2A	Silence target expression by AGO-mediated RNA cleavage	Esophageal cancer	37317053
	SRSF3	siRNA	SRSF3	Silence target expression by AGO-mediated RNA cleavage	Pancreatic cancer	35545048
	SRSF6	siRNA	SRSF6	Silence target expression by AGO-mediated RNA cleavage	Colorectal cancer	29114070
	SRSF7	siRNA	SRSF7	Silence target expression by AGO-mediated RNA cleavage	Glioblastoma	34954129
Decoy RNA Oligonucleotides	SRSF1	SF2i1 and SF2i2	MKNK2-p38-MAPK	Affect SR by directly binding the RRM domains	Glioblastoma	30962446

### Repurposing chemicals from approved drugs

Since the early findings of inhibitors that alter HIV-1 splicing via SR proteins, attempts have been focused on screening for new antiviral compounds [106]. These results are surely valuable for viral-induced cancer treatment. For cancers independent of viral infection, therapeutic strategies targeting SR proteins have also been actively developed in recent years. SRSF6 is frequently amplified and upregulated in several cancers, including colon, lung, and breast cancer [107]. On the basis of the predicted three-dimensional (3D) structure from homology modeling, Wan et al. virtually screened compounds in the DrugBank database and identified indacaterol as a potent inhibitor targeting the RRM2 domain of SRSF6. Indacaterol is known as a β2-adrenergic receptor agonist approved for chronic obstructive pulmonary disease treatment. Functional experiments showed that indacaterol can inhibit cancer progression in colorectal tumor cells in a murine xenograft model [108]. Inspired by this preliminary study, we utilized indacaterol as a structural template to select nebivolol, a β1-adrenergic receptor antagonist, as an RRM domain antagonist of TRA2A. Follow-up experiments confirmed that nebivolol can compete with RNA targets of TRA2A to interfere with its downstream signaling in esophageal cancer cells [98]. Similarly, potent compounds have also been repurposed for SRSF3 inhibition [109].

However, SR proteins harbor similar structural domains and often cross-regulate each other or autoregulate itself [76, 110]. For example, the expression levels of SRSF10 expression correlate with other SR proteins, and depletion of SRSF10 autoregulation affects the expression of all SR proteins [110]. Thus, it needs to be investigated whether there are profound effects on other SR proteins when testing the compounds. Indeed, there is a report that GPS167/192, which impacts the activity of SRSF10, demonstrated a modest but statistically significant effect on other SR proteins [111].

### Designer RNA-based therapeutic agents

Effective compound optimization needs the exact protein crystal structure, which is usually limited for SR family proteins. As an alternative approach, RNA oligonucleotides, which solely depends on the sequence information and can be easily synthesized, have shown promising results on the modulation of splicing via SR proteins. We categorize these kinds of inhibitors in the following three classes: small interfering RNAs (siRNAs), antisense oligonucleotides (ASOs), and decoy RNAs.

(A) Small interfering RNAs (siRNAs) are synthetic double-stranded RNAs and can induce target RNA cleavage via AGO2. They have long been used in research to downregulate endogenous SRSFs, such as SRSF3, SRSF6, SRSF7, and TRA2A [93, 96, 108]. However, the clinical application of siRNA to SR proteins is still not possible owing to off-target effects and lack of efficiency in in vivo delivery methods.

(B) Antisense oligonucleotides (ASOs) are short, single-stranded oligonucleotides that trigger different mechanisms, such as RNA degradation, altered splicing, and translational arrest, to modulate target transcript expression [112]. As previously described, inclusion of a PE triggers auto downregulation of SR protein expression via a NMD pathway. Members of the SR family can bind to the PE-splicing regulatory sequences, therefore allowing SR proteins to compete or cooperate to regulate the AS of PE. Based on this principle, ASOs targeting the regulatory sequence to promote or reduce PE inclusion have achieved successful control of TRA2B and SRSF3 expression, suggesting that such splice switching oligomers can be potential anticancer drugs [76, 113].

(C) Denichenko et al. synthesized three tandem motif repeats against the RRM domain of SRSF1 but not resemble the known ESE [114]. Experiments have shown these RNA decoys directly bind SRSF1 and affect splicing. The main advantage of this approach is that decoy oligonucleotides affect SR protein-RNA binding activities without disturbing the interaction between SR protein and other proteins, thus providing target selectivity and less toxicity than complete knockdown of SR protein.

Although RNA oligonucleotides can target specific SR proteins, there is still the possibility that siRNAs, ASOs, or decoy RNAs may cause dysregulation of AS events in multiple downstream molecules owing to the ability of one SR protein to regulate multiple AS events. To further enhance selectivity and to reduce toxicity, it is expected that treatment to control gene-specific splicing events without globally affecting cellular splicing will be developed. This would be achieved by carefully disentangling the specific interactions between splicing sites and the domains in SR proteins under disease conditions, and by utilizing a targeted approach, i.e., CRISPR/Cas9-mediated deletion, to modify the regulatory binding sequence of SR proteins. Moreover, it is necessary to assess the inhibitory effects by setting proper normal controls to ensure that the cancer cells are sensitive to subtle AS changes induced by the compounds while the normal cells can tolerate such alteration.

### Developing treatments based on novel mechanisms

Detailed mechanistic studies have found additional clues for SR protein regulation. For instance, Zhou et al. found a significant increase of SRSF transcripts, including SRSF6, in T-cell acute lymphoblastic leukemia (T-ALL) compared with normal T cells [115]. Furthermore, they found that USP7 controls SRSF6 degradation via deubiquitination. Thus, proteasome inhibitors could be exploited for therapeutic use to control SR protein expression and inhibit T-ALL growth. Similar ubiquitylation-mediated control of protein degradation has been found for SRSF3, SRSF6, and TRA2A [115–117]. Furthermore, TRA2A is transcriptionally induced by hypoxia-inducible factor 1 subunit alpha (HIF1α) in pancreatic cancer [118]. Thus, these regulatory mechanisms can be developed into novel treatments in the future.

## Conclusions

In this review, we summarize the canonical and noncanonical functions of SR proteins during carcinogenesis. In the future, more efforts are expected to reveal the functional significance of SR proteins and the relevant pathways during cell transformation. To translate the mechanistic findings of SR proteins into the clinic, novel drug developmental strategies are also needed to improve the specificity, safety, and efficiency in modulating the SR proteins and gene splicing, which will ultimately turn SR proteins into actionable therapeutic targets in cancer.

## Data Availability

Not applicable.

## Abbreviations

SR protein: Serine and arginine-rich protein
RRM: RNA recognition motif
ncRNA: Non-coding RNA
AS: Alternative splicing
APA: Alternative cleavage and polyadenylation
NMD: Nonsense-mediated decay
SRPK: SR protein kinase
CLK: Cdc2-like kinase
DYRK: Dual-specificity tyrosine phosphorylation regulated kinase
U2AF: U2 auxiliary factor
5′SS: 5′ Splice sites
3′SS: 3′ Splice sites
ESE: Exonic splicing enhancer
ESS: Exonic splicing silencer
siRNA: Small interfering RNA
